# A case of papillary hyperplasia of thyroid misdiagnosed as papillary thyroid carcinoma: Case report and review of literature

**DOI:** 10.1002/ccr3.4867

**Published:** 2021-11-07

**Authors:** Sara Faisal Alharbi, Arwa Ahmed Abba, Amal Abdulghaffar Hafiz, Aroob Mohammedamin Abdulhalim, Gadir Yaqoub Bokhari, Ghada Elsayed Esheba

**Affiliations:** ^1^ Department of Pathology College of Medicine Umm Al‐Qura University Makkah Saudi Arabia; ^2^ Faculty of Medicine Department of Pathology Tanta University Tanta Egypt

**Keywords:** CD56, CK19, galectin‐3, immunohistochemical markers, mesothelial antigen, NGAL, papillary thyroid carcinoma (PTC), papillary thyroid hyperplasia

## Abstract

Overdiagnosis of papillary thyroid hyperplasia which may mimic papillary thyroid carcinoma in fine‐needle aspiration and frozen section has a serious impact on the patient. Therefore, it is important to know the difference between them to avoid over or undertreatment.

## Graphical abstract

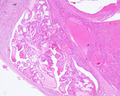

## 1 INTRODUCTION

We present a case of papillary thyroid hyperplasia that was misdiagnosed as papillary thyroid carcinoma on fine‐needle aspiration and as a consequence, the patient underwent left hemithyroidectomy. Histopathology revealed encapsulated nodule with papillae, focal nuclear clearing, and occasional grooves. Immunohistochemistry showed negative expression for CK19, Galectin‐3, NGAL markers, mesothelial antigen, and strong staining for CD56.

Papillary thyroid hyperplasia (PTH) may mimic papillary thyroid carcinoma (PTC) in fine‐needle aspiration cytology (FNAC) and frozen section, therefore, it may be misdiagnosed as PTC causing over treatment by lobectomy or total thyroidectomy which has a serious impact on patient's prognosis. Histopathologically, papillary hyperplasia usually presents with a well‐circumscribed encapsulated nodule, sometimes with cystic changes centrally. The nuclei are round, with occasional nuclear grooves, Psammoma bodies, and calcification could be present as well.^1^, ^2^

The gold standard method for the diagnosing of PTC is by using eosin staining.^2^ The main diagnostic histopathological features are the presence of characteristic nuclear features such as nuclear enlargement and elongation, nuclear clearing or ground glass nuclei, grooves, pseudoinclusions, overlapping, and atypia. Another diagnostic feature is Psammoma bodies which are not seen frequently in benign lesions.^2^, ^3^ Immunohistochemistry could differentiate between PTC and PTH in challenging cases, where the expression of CK19, galectin‐3, NGAL, D2‐40, vascular endothelial growth factor (VEGF), epidermal growth factor receptor (EGFR) are positive in most cases of PTC. These biomarkers are proven to be the most sensitive and specific markers for the diagnosis of PTC. On the other hand, the expression of these markers is completely negative in cases of PTH. On the other hand, CD56 is expressed in normal thyroid follicular cells and is preserved in benign thyroid lesions, while it is absent in PTC.^2^, ^4^

Thyroid hyperplasia is managed by the use of an antithyroid drug (ATD) or radioactive iodine (RAI) treatment or less commonly total thyroidectomy in cases of large goiter.^5^ In this study, we report a case of PTH that was misdiagnosed as PTC during FNA and the patient underwent a left hemithyroidectomy resulting from the overdiagnosis. Also, we highlight the importance of differentiation between PTH and PTC to avoid overtreatment of the patients.

## 2 CASE PRESENTATION

A 32‐year‐old female presented with swelling in the region of the thyroid of 9 months duration. Clinical examination was suggestive of multinodular goiter and there was no evidence of symptoms indicative of hypothyroidism or hyperthyroidism. Otherwise, she had no notable medical history. The serum thyroid profile revealed euthyroid status. Ultrasound revealed an oval solid nodule with heterogeneous echogenicity in the left upper pole of the thyroid. Most of the nodule was isoechogenic, but there was a hypoechoic focus within the nodule. The nodule size was 0.7 × 0.5 cm.

**FIGURE 1:**
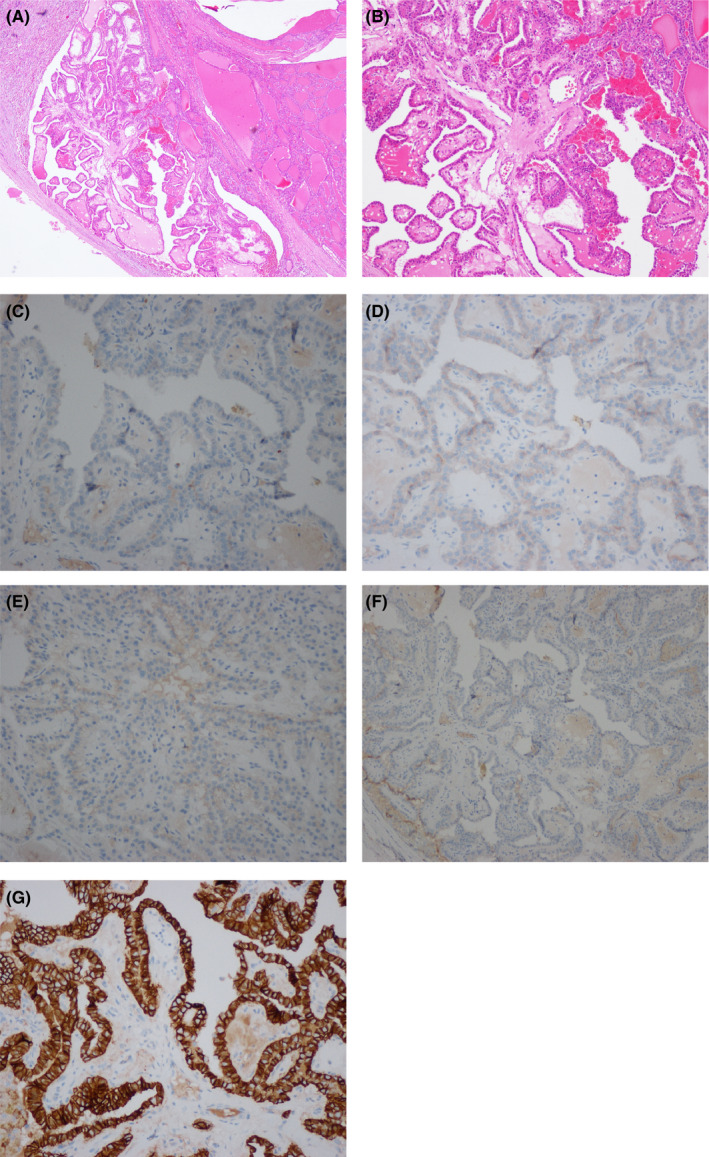
(A) Well‐circumscribed nodule with papillary formation (H&E ×40). (B) PTH shows papillae that lack the characteristic nuclear features of classic PTC (×100). (C) Negative expression for CK19 (×200). (D) Negative expression for galectin‐3 (×200). (E) Negative staining for NGAL (×200). (F) Negative staining for mesothelial antigen. (×100). (G) Strong complete membranous staining for CD56 (×200)

U/S‐guided FNAC was done in a different hospital and revealed the presence of papillae lined by epithelial cells with enlarged nuclei and occasional grooves, which was suspicious for PTC (Bethesda system category V). The patient underwent a left hemithyroidectomy. Macroscopically, the cut surface of the thyroid showed a well‐circumscribed encapsulated gray tan mass with a focal whitish solid area. Histologically, the nodule was composed of thyroid follicles and surrounded with a thin fibrous capsule, which favored follicular adenoma. A focal area of papillary architecture lined by follicular cells with enlarged nuclei and rare nuclear grooves were seen (Figure 1A,B). The slides were referred to our department as a consultation case. We revised the slides and performed an immunohistochemical panel which revealed negative expression of the lining epithelial cells for CK19 (Figure 1C), Galectin‐3 (Figure 1D), NGAL (Figure 1E), and mesothelial antigen (Figure 1F). On the other hand, the lining cell showed strong complete membranous staining for CD56 (Figure 1G) which favored PTH. Thus, our patient was diagnosed as PTH by the features of the histopathology and negative immunohistochemical staining for CK19, Galectin‐3, NGAL, mesothelial antigen, and positive staining for CD56.

The diagnosis was made as follicular adenoma with focal papillary hyperplasia.

The patient was followed up for 6 months after the surgery, and she was symptom‐free.

## 3 DISCUSSION

Here, we report a case of PTH that was misdiagnosed as PTC. Our patient presented with abnormal neck swelling in the thyroid region for 9 months duration with no evidence of symptoms indicative of hypothyroidism or hyperthyroidism. The patient underwent a left hemithyroidectomy in another institution, and then the slides were referred to our department as a consultation case. The differential diagnosis of this case besides papillary thyroid carcinoma includes hyperplasia (diffuse and adenomatous), Hashimoto's disease, autoimmune thyroiditis, and some adenomas particularly trabecular type.^2^

Microscopic examination showed encapsulated and well‐circumscribed nodule with papillae lined by epithelial cells that have round nuclei with occasional nuclear grooves.

Papillary thyroid hyperplasia may be confused with PTC based on FNAC, resulting in overtreatment either by lobectomy or by total thyroidectomy, depending on clinical parameters such as the size of the nodule and other risk factors, sonographic findings, and patient preference.^1^ There are challenging features in histopathology that result in potential diagnostic difficulty includes the presence of well‐developed papillary fronds with fibrovascular cores and nuclear pleomorphism in both PTH and PTC.^3^

There are only a few published reports on the diagnostic dilemma of differentiating PTH from PTC by histopathology.^3^ Similar to our case, Flikweert et al have reported a case of a 40‐year‐old woman with swelling in the left lobe of the thyroid gland. The case was suspected to be papillary thyroid carcinoma based on fine‐needle aspiration cytology and frozen section examination. Hence, total thyroidectomy plus cervical‐central lymph node dissection was performed. However, the ultimate histological examination revealed papillary hyperplasia rather than papillary thyroid carcinoma.^6^

Marc P. Pusztaszeri et al conducted a retrospective study aimed to compare the causes of diagnostic error of PTH and PTC by histopathology. The study included 48 cases of PTH and 50 cases of PTC. Cytological examination of the PTC cases showed enlarged nuclei with nuclear pallor, numerous grooves, cellular swirls, psammoma bodies, and pseudoinclusions which were not seen in the benign thyroid nodule with papillary hyperplasia (BTN‐PH). On the other hand, cases of BTN‐PH demonstrated mild nuclear atypia and rare nuclear grooves. However, no intranuclear pseudoinclusions were detected. The authors also found psammomatous calcifications in the 4 cases of BTN‐PH.^1^

Khayyata et al also reported a similar diagnostic challenge in interpreting hyperplastic nodules during cytology as suspicious for or consistent with PTC. The latter authors also mentioned that benign papillary lesions are consisting of short, poorly formed, non‐branching papillae with avascular cores containing loosely arranged spindle cells. On the contrary, the pathognomonic feature of PTC is branching papillae with fibrovascular cores. Similar to our case, intranuclear grooves were occasionally detected in papillary hyperplastic nodules, nevertheless, there are no other features of papillary carcinoma.^7^

## 4 CONCLUSION

The data from this case report highlights the importance of CK19, galectin‐3, NGAL, and CD56 as potentially helpful immunohistochemical markers in differentiating papillary thyroid hyperplasia from papillary thyroid carcinoma in equivocal cases in addition to careful microscopic examination, which help in reducing diagnostic errors and improve the patient's outcome.

## CONFLICTS OF INTEREST

The authors declare no potential conflicts of interest with respect to the research, authorship, and/or publication of this article.

## AUTHOR CONTRIBUTIONS

All authors made substantial contributions to conception, design of the study, took part in drafting the article or revising it critically for important intellectual content, agreed to submit to the current journal, gave final approval of the version to be published, and agree to be accountable for all aspects of the work.

## ETHICAL APPROVAL

The current study was approved by the ethics committee at the faculty of medicine at Umm Al‐Qura University. Written consent was obtained from the patient to publish.

## CONSENT

Published with written consent of the patient.

## Data Availability

The data that support the findings of this study are available from the corresponding author upon reasonable request.
